# Subliminal Impending Collision Increases Perceived Object Size and Enhances Pupillary Light Reflex

**DOI:** 10.3389/fpsyg.2016.01897

**Published:** 2016-12-02

**Authors:** Lihong Chen, Xiangyong Yuan, Qian Xu, Ying Wang, Yi Jiang

**Affiliations:** ^1^State Key Laboratory of Brain and Cognitive Science, CAS Center for Excellence in Brain Science and Intelligence Technology, Institute of Psychology, Chinese Academy of SciencesBeijing, China; ^2^University of Chinese Academy of SciencesBeijing, China

**Keywords:** threat, looming, size perception, pupillary light reflex, awareness

## Abstract

Fast detection of ambient danger is crucial for the survival of biological entities. Previous studies have shown that threatening information can bias human visual perception and enhance physiological reactions. It remains to be delineated whether the modulation of threat on human perceptual and physiological responses can take place below awareness. To probe this issue, we adopted visual looming stimuli and created two levels of threat by varying their motion trajectories to the observers, such that the stimuli could move in a path that either collided with the observers’ heads or just nearly missed. We found that when the observers could not explicitly discriminate any difference between the collision and the near-miss stimuli, the visual stimuli on the collision course appeared larger and evoked greater pupil constrictions than those on the near-miss course. Furthermore, the magnitude of size overestimation was comparable to when the impending collision was consciously perceived. Our findings suggest that threatening information can bias human visual perception and strengthen pupil constrictions independent of conscious representation of the threat, and imply the existence of the subcortical visual pathway dedicated to automatically processing threat-related signals in humans.

## Introduction

The ability to quickly detect and properly react to potential dangers in the environment is of evolutionary significance to living organisms. One may have to judge whether a looming ball will hit him/her in order to make a proper response (e.g., to avoid collision). Threat-related signals have been found to bias human visual perception and trigger physiological responses. For example, people are inclined to overestimate the perceived proximity (Cole et al., 2013), size (Vasey et al., 2012), and duration (Tipples, 2011) of threatening objects, or to underestimate the time to contact with them (Vagnoni et al., 2012). Observers with fear of heights overestimate the perceived vertical distances and the sizes of objects when looking down from a high place (Teachman et al., 2008; Stefanucci and Proffitt, 2009). Furthermore, pictures of threatening stimuli elicit larger physiological reactions, including enhanced pupil constrictions and skin conductance responses (SCRs) in comparison with non-threatening ones (Naber et al., 2012; Lapate et al., 2014).

As a type of dynamic threatening information, visual looming stimuli have been observed to evoke the escape responses in a variety of species (Schiff et al., 1962; Wang and Frost, 1992; de Vries and Clandinin, 2012; Yilmaz and Meister, 2013; Temizer et al., 2015), including human infants (Ball and Tronick, 1971). A growing body of evidence suggests that the threat content (e.g., impending collision) conveyed by the looming stimuli biases human visual perception and captures attention. For example, the looming stimuli predominate over receding ones during binocular rivalry (Parker and Alais, 2007), and they appear larger than the contracting ones (Whitaker et al., 1999). Moreover, the looming objects on a collision course with the observers capture attention more strongly than those on a near-miss course (Lin et al., 2008).

It remains to be delineated whether the modulation effects of looming stimuli on visual perception and physiological reactions have to rely on the conscious representation of the threatening information. Some previous studies provide clues to this issue. For instance, the attentional effect induced by impending collision can be observed without observers’ explicit discrimination between the collision objects and the near-miss ones (Lin et al., 2009). It would be reasonable to postulate that the modulation of looming stimuli on visual perception and physiological reactions might be automatic and independent of whether the threat elicited by the looming stimuli is consciously perceived and explicitly discriminated. To probe this issue, we used very brief looming stimuli and created two levels of threat by varying the motion trajectories of the looming stimuli, such that the effects evoked by the two types of motion trajectories, one collided with the observers’ heads and the other nearly missed, could be directly compared. Since the collision stimuli contained more threatening information than the near-miss ones, it would be expected that the former would bias visual size perception more strongly and evoke greater physiological reactions than the latter. The more critical examination was whether the perceptual and physiological effects would persist even when the observers could not consciously discriminate the collision stimuli from the near-miss ones.

## Materials and Methods

### Participants

A total of 44 undergraduates and graduates (22 male) with the mean age of 22.25 (ranging from 19 to 26) received financial compensation for their participation. All the information about participants, stimuli, and tasks was summarized in **Table 1**. In Experiment 1, 10 observers (5 male) participated in the size perception and motion trajectory discrimination tasks. In Experiment 2, 12 observers (6 male) performed the size perception and motion trajectory discrimination tasks. In Experiment 3, another 8 observers (4 male) participated in the two-alternative forced choice (2AFC) tasks on motion trajectory and speed discrimination. In Experiment 4, a new group of 8 observers (4 male) participated in the distance discrimination and the corresponding 2AFC tasks (one of them also participated in Experiment 1). Seven observers (3 male) participated in Experiment 5, which includes size perception, motion trajectory discrimination and location discrimination tasks (with their pupil sizes recorded). All had normal or corrected-to-normal vision and gave written, informed consent in accordance with procedures and protocols approved by the institutional review board of the Institute of Psychology, Chinese Academy of Sciences. All observers were naive to the purpose of the experiments.

**Table 1:** Summarized experimental settings.

Experiment	Participants	Stimuli	Tasks
1	10	3/6 cm final impact points (gray background)	size perception, motion trajectory discrimination
2	12	4/6 cm final impact points (gray background)	size perception, motion trajectory discrimination
3	8	4/6 cm final impact points (gray background)	2AFC motion trajectory discrimination, 2AFC speed discrimination
4	8	4/6 cm final impact points (gray background)	distance discrimination, 2AFC distance discrimination
5	7	4/6 cm final impact points (black background)	size perception, motion trajectory discrimination, location discrimination (with eye tracking)

### Apparatus, Stimuli, and Procedure

Subjects viewed a CRT monitor binocularly from a distance of 50 cm. A chin rest was used to stabilize their head position. The experimental room had no illumination other than the display screen. Displays were generated using Matlab (Mathworks) together with the Psychophysics Toolbox (Brainard, 1997; Pelli, 1997).

**FIGURE 1:**
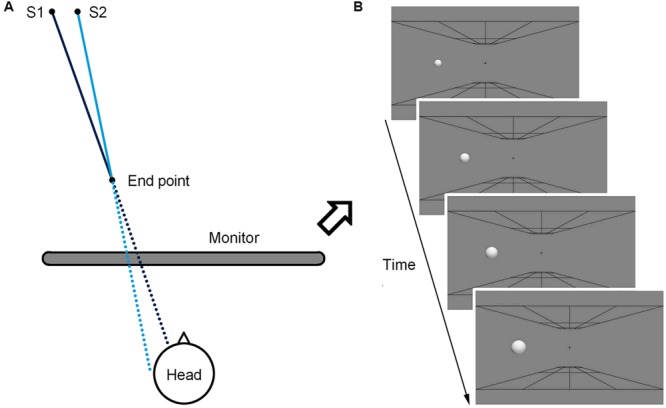
**Illustrations of the simulated motion trajectories of the spheres in three-dimensional coordinates (A)** and on the screen **(B)**. The dark blue line represents the collision path and the light blue line represents the near-miss path. The solid lines are the simulated trajectories of the spheres moving from 350 cm to 150 cm from the observers, and the dashed lines are the imaginary trajectories if the spheres continue to move after stillness. S1 and S2 are the simulated start locations of the collision and the near-miss spheres, respectively.

The looming sphere simulated a motion from a distance of 350 cm to 175 cm to the observers (**Figure 1A**), and during this period it expanded uniformly from a small size (1.32°) to the standard size (2.64°; **Figure 1B**). In Experiment 1, the motion duration was 133 ms. The collision sphere had an initial position 7.57 cm (8.68°) from the monitor center and simulated a final impact point 3 cm from the center of the observers’ heads. Similarly, the near-miss sphere had an initial position 7.14 cm (8.18°) from the monitor center and simulated a final impact point 6 cm from the center of the observers’ heads. At the end of the looming motion, both the collision and the near-miss spheres had identical end position 8 cm (9.17°) from the monitor center. In Experiments 2–5, two parameters were altered to render the trajectory difference indistinguishable. Firstly, the motion duration was reduced to 67 ms. Secondly, the initial position of the collision sphere was 7.43 cm (8.51°) from the monitor center, simulating a point of impact 4 cm from the center of the observers’ heads.

In the size perception task, the sphere stayed on the screen for an extra 200 ms after it reached its maximum size, and then followed by a comparative circle presented at the center of the screen. The observers were asked to adjust the size of the comparative circle to match the final size of the sphere presented before. The perceived size of the sphere was calculated relative to its standard size. The initial size of the comparative circle varied from trial to trial (2.40°–2.87°) with a step of 0.068°. In the motion trajectory discrimination task, one collision or near-miss sphere was presented in each trial. The observers were asked to judge whether the sphere collided with their heads or just nearly missed (subjective judgment task). In 2AFC tasks, two spheres were presented successively, one on the collision path, and the other on the near-miss path. The observers had to judge whose trajectory collided with their heads, the first one or the second one in the 2AFC motion trajectory discrimination task, and to judge which one was faster or nearer to them in the 2AFC speed discrimination and the 2AFC distance discrimination tasks, respectively. In another task of distance discrimination, the observers had to judge whether the successively presented spheres had the same or different distances from them.

### Eye Tracking

We only recorded the observers’ pupil sizes in Experiment 5 where the observers were asked to perform the location discrimination task. To exclude the influence of the physical difference of motion trajectories, we included another two conditions in which the spheres moved in the same trajectories as in the collision and near-miss conditions, but from the end point to the starting point, i.e., in a receding manner. Because of the extreme sensitivity of the pupil to the light, we replaced the gray background with a black one in Experiment 5. In each trial, after a variable duration (1500–1900 ms) with a white fixation cross presented at the center of the screen, a looming or receding sphere was presented to the left or right side of the fixation cross with a duration of 67 ms, following by a 4-s blank interval. The observers were asked to judge the location of the sphere relative to the fixation cross using the left and right arrow keys. To maintain an accurate measure of the pupil size, the observers were required to keep their eyes on the fixation cross and to refrain from blinking throughout the trial.

Pupil diameter and two-dimensional eye position of the left eye were measured with a video-based iView X Hi-Speed system (SMI, Berlin, Germany). A standard 5-point calibration was performed at the beginning of each session. Eye tracking data were acquired at 500 Hz. Trials with unrealistic pupil size (<0.3 mm or >1.3 mm) and with the pupil size out of ±3 SDs were excluded from further analysis. There were 20.25% trials on average being excluded in the eye tracking experiment. The data were resampled in time bins of 40 ms each. For each subject and each condition, pupil data were averaged across trials after subtracting the mean pupil diameter in the 500 ms preceding stimulus onset. Horizontal eye position data were analyzed in the same way as the pupil diameter.

There were 80 trials in each of the 2AFC trajectory discrimination task, 2AFC speed discrimination task and the two distance discrimination tasks. There were 32 trials per condition in the remaining tasks.

## Results

### Threatening Information Increases Perceived Object Size Independent of Conscious Perception

**FIGURE 2:**
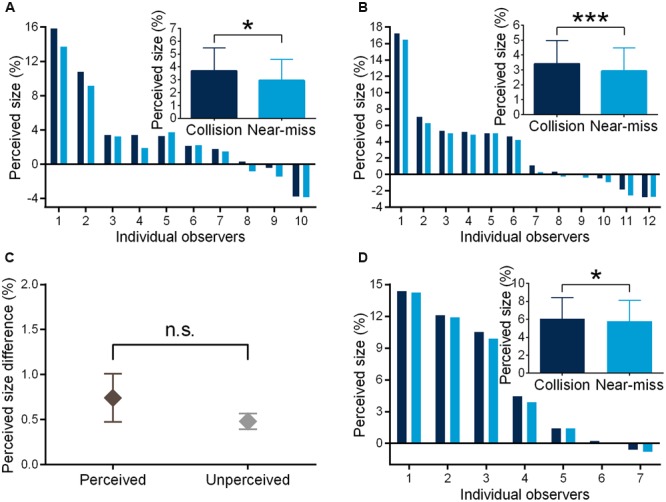
**Perceived size of the looming spheres in the threat-perceived (A)** and -unperceived **(B)** conditions, respectively. **(C)** The perceived size discrepancies between the collision and near-miss spheres were similar in the two conditions. Perceived size of the looming spheres with black background in the threat-unperceived condition **(D)**. ^∗^*p* < 0.05, ^∗∗∗^*p* < 0.001. The error bars represent one standard error of the mean. The perceived size of the sphere was calculated relative to its standard size.

In Experiment 1, the observers could well discriminate the collision sphere from the near-miss one (judgment accuracy = 62.1% ± 8.0%, 95% Confidence Interval (CI) = [56.3%, 67.8%], *t*(9) = 4.757, *p* = 0.001, *d* = 1.504, JZS-BF [alternative/null] = 44.553, mean sensitivity *d*′ = 0.683 ± 0.532). JZS-BF is Jeffrey-Zellner-Siow Bayes factor with Cauchy distribution on effect size and is the probability of the data under one hypothesis relative to that under another hypothesis (Rouder et al., 2009; Vadillo et al., 2016). The odds of alternative versus null hypothesis were greater than 44:1 favoring the alternative hypothesis. The collision sphere was perceived to be significantly larger than the near-miss one (3.682% vs. 2.941%, *F*(1,9) = 7.688, *p* = 0.022, η_p_^2^ = 0.461; **Figure 2A**). In Experiment 2, the observers could not discriminate the trajectory difference any more (50.5% ± 5.4%, 95% CI = [47.1% 54.0%], JZS-BF [null/alternative] = 4.421, mean sensitivity *d*′ = 0.036 ± 0.339). The odds of null versus alternative hypothesis were greater than 4:1 favoring the null hypothesis. Remarkably, the collision sphere still appeared larger than the near-miss one (3.409% vs. 2.928%, *F*(1,11) = 30.858, *p* < 0.000, η_p_^2^ = 0.737; **Figure 2B**). In Experiment 3, to further confirm the results of Experiment 2, we asked another group of the observers to perform a 2AFC motion trajectory discrimination task, and found that the observers could not discriminate the trajectory difference (53.8% ± 8.4%, 95% CI = [46.7%, 60.8%], JZS-BF [null/alternative] = 1.987). Moreover, the discrepancy of perceived size in Experiment 2 could not be explained by the differences in speed perception between the two conditions (52.0% ± 7.7%, 95% CI = [45.6%, 58.5%], JZS-BF [null/alternative] = 3.033,). In Experiment 4, we found that distance perception could not account for the discrepancy of perceived size between the two conditions in Experiment 2 (2AFC performance, 52.2% ± 6.5%, 95% CI = [46.8% 57.6%], JZS-BF [null/alternative] = 2.579; same-difference judgment, 50.2% ± 8.7%, 95% CI = [42.9%, 57.4%], JZS-BF [null/alternative] = 3.910). More interestingly, the perceived size discrepancy of the collision and near-miss spheres in Experiment 1 and Experiment 2 was not different from each other [*F*(1,20) = 0.997, *p* = 0.330, η_p_^2^ = 0.047, **Figure 2C**], which is in contrast to the observations that the observers’ motion trajectory discrimination accuracies in Experiment 1 was significantly higher than those in Experiment 2 [*F*(1,20) = 16.099, *p* = 0.001, η_p_^2^ = 0.446]. Moreover, the observers’ size overestimation effects were not correlated with their motion trajectory discrimination accuracies [*r*(22) = 0.248, *p* = 0.265]. These results together suggest that the modulation effect of threat on perceived size is automatic and independent of conscious perception.

### Subliminal Threat Enhances Pupil Constriction

In Experiment 5, we first replicated the behavioral finding that the observers could not discriminate the motion trajectory difference (53.6% ± 10.6%, 95% CI = [43.8% 63.4%], JZS-BF [null/alternative] = 2.593, mean sensitivity *d*′ = 0.296 ± 0.756). The odds of null versus alternative hypothesis were around 3:1 favoring the null hypothesis. The collision sphere was perceived as significantly larger than the near-miss one (6.078% vs. 5.792%, *F*(1,6) = 11.228, *p* = 0.015, η_p_^2^ = 0.652; **Figure 2D**).

**FIGURE 3:**
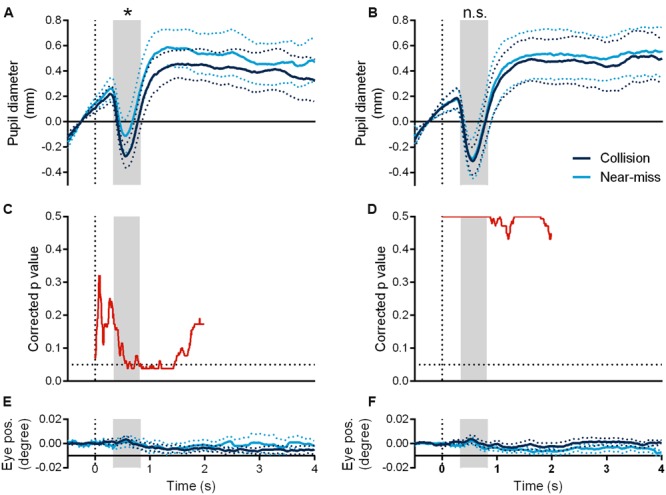
**Time courses of pupil diameters in the looming (A)** and receding conditions **(B)** and their corresponding horizontal eye gaze positions **(E,F)**. Corrected *p*-value of permutation test of pupil diameter discrepancy between the collision and the near-miss spheres in the two conditions **(C,D)**, respectively. The gray area indicates the time duration ranging from 340 ms to 820 ms after the onset of the sphere. ^∗^*p* < 0.05. The dotted lines represent one standard error of the mean.

The pupil diameters of the observers began to shrink around 300 ms after the onset of the sphere. We found a marginally significant interaction of presentation mode (looming and receding) and motion trajectory (collision and near-miss) in the minimum vertex of pupil diameters [*F*(1,6) = 5.601, *p* = 0.056, η_p_^2^ = 0.483]. Further analysis showed that the collision sphere evoked significantly larger pupil constrictions than the near-miss one in the looming manner (*t*(6) = –3.309, *p* = 0.016, *d* = –1.251, JZS-BF [alternative/null] = 4.769, **Figure 3A**). However, there was no such difference when the spheres moved in the receding manner (*t*(6) = –0.230, *p* = 0.826, JZS-BF [null/alternative] = 3.613, **Figure 3B**), even though the receding spheres had the identical motion trajectories as the looming spheres. This pattern of results persisted from 340 ms to 820 ms after the onset of the sphere, as evidenced by a significant interaction between presentation mode (looming and receding) and motion trajectory (collision and near-miss), *F*(1,6) = 5.991, *p* = 0.05, η_p_^2^ = 0.50. Consistently, the collision sphere elicited significantly larger pupil constrictions than the near-miss one in the looming manner (*t*(6) = –3.242, *p* = 0.018, *d* = –1.225, JZS-BF [alternative/null] = 4.446) but not in the receding manner (*t*(6) = –0.238, *p* = 0.820, JZS-BF [null/alternative] = 3.606). Differences in the pupil diameters between the collision and near-miss spheres were also tested for statistical significance through one-tailed non-parametric permutation test based on the t-max statistic (Blair and Karniski, 1993; Groppe et al., 2011). We performed the test in the time window of 0 ms to 2000 ms after the onset of the sphere (5000 permutations, *p* = 0.05). The results showed that the collision sphere elicited significantly larger pupil constrictions than the near-miss one from 570 ms to 1460 ms after the onset of the sphere in the looming manner (**Figure 3C**), but not in the receding manner (**Figure 3D**). Furthermore, the difference of pupil diameters between the collision and the near-miss spheres in the looming manner was not caused by horizontal eye gaze difference (*t*(6) = 0.248, *p* = 0.812, JZS-BF [null/alternative] = 3.598; **Figure 3E**; see **Figure 3F** for the receding manner).

## Discussion

Previous studies have demonstrated that consciously perceived threatening information can bias human visual perception in both spatial and temporal domains. To probe the modulation of subliminal threat on visual size perception and pupillary light reflex, we used visual looming stimuli and created two levels of threat by varying their motion trajectories in three-dimensional geometric space, such that it could either collide with the observers’ heads or just nearly miss. We found that, regardless of whether or not the observers were aware of the differences between the collision and the near-miss spheres, the former was perceived as significantly larger than the latter, and the effect of size overestimation was comparable between the threat-perceived and the threat-unperceived conditions. Moreover, the subliminal threat induced by impending collision elicited greater pupil constrictions. These results provide evidence that visual looming stimuli can modulate visual perception and physiological reactions in an automatic fashion and independent of whether or not the threat elicited by impending collision of looming stimuli is consciously perceived.

Converging evidence has shown that size perception can be biased by threatening information. For example, circles with a negative picture are estimated to be larger than circles with a positive or a neutral picture (van Ulzen et al., 2008). Moreover, observers with fear of heights tend to overestimate object size when looking down from a high place (Stefanucci and Proffitt, 2009), and those with spider phobia often overestimate the sizes of spiders (Vasey et al., 2012; Shiban et al., 2016). Visual looming stimulus that implies impending collision looks larger than a receding one (Whitaker et al., 1999). However, all these effects are observed when the observers are fully aware of the potential threat. The current study provides further evidence and shows that even when the threatening information is not consciously identified, it can still bias object size perception, and the amplitude of such misperception is comparable to the threat-perceived condition. Previous studies have demonstrated that threatening information, such as a visual stimulus paired with white noise (Koster et al., 2004) or a looming stimulus on a collision path (Lin et al., 2008, 2009), can automatically attract attention, and allocation of spatial attention onto a visual stimulus can affect its perceived size (Anton-Erxleben et al., 2007). Therefore, it seems possible that the effects of threat on size perception and attentional capture work in tandem to enhance the salience of the threatening information and guide the observers to make further actions.

Although the current study does not provide direct evidence on the possible mechanisms underlying the observed effects, it is postulated that a specialized subcortical visual pathway (through the superior colliculus and the pulvinar to the amygdala) is dedicated to detecting threat-related signals outside of conscious awareness (Hedger et al., 2015). This idea has been supported by converging evidence showing that subliminal threat-related visual stimuli elicit enhanced neural and physiological responses in comparison with non-threat stimuli. For example, fearful faces and spider images have been shown to enhance the amygdala activity in the absence of visual awareness across a variety of masking techniques (Whalen et al., 1998; Pasley et al., 2004; Williams et al., 2004; Jiang and He, 2006; Lipka et al., 2011), likely through the purported subcortical pathway (Tamietto and De Gelder, 2010). Visual looming stimuli are often associated with imminent collision and considered as potent perceptual indicators of threat. By using the visual looming stimuli that simulated impending collision with observers’ heads to elicit threat, the current study resonates well with and extends previous findings by showing that the threatening information can be processed independent of conscious awareness, and thus suggests the existence of the subcortical visual pathway dedicated to processing threat-related signals independent of conscious perception in humans.

It is widely accepted that adjustments of pupil size occur automatically in response to variations in ambient light to optimize retinal illumination for visual perception. However, recently a growing body of evidence has shown that the pupil not only constricts to physically bright object, but also to bright illusions (Laeng and Endestad, 2012), and even to imaginary bright situations (Laeng and Sulutvedt, 2014). Moreover, pupil constrictions as well respond to a variety of visual attributes besides brightness, including the inversion effect (Conway et al., 2008; Naber and Nakayama, 2013), novelty (Naber et al., 2013), conspecific faces (Conway et al., 2008; Ebitz et al., 2014), threatening animals (Naber et al., 2012), and the sun (Binda et al., 2013; Naber and Nakayama, 2013). The current study extends the scope of stimulus properties the pupil responds to, and demonstrates that it also constricts to subliminal threatening content conveyed by impending collision. This response pattern should have adaptive value for survival, because it suggests that the brain already processes the threatening signals even before we consciously realize them, which might enable humans to act fast in fight or flight situations. More importantly, the combined evidence from previous studies and the current one suggests that pupil constrictions are not merely reflexive responses to retinal illumination, but are also mediated by the automatic visual processing of salient events in the environment. It has been suggested that the pupillary light responses are largely mediated by subcortical non-image forming system (Binda and Murray, 2015), and the retinocollicular pathway directly connecting the retina to the superior colliculus and the pulvinar is engaged in the eye movements in the absence of awareness (Spering and Carrasco, 2015), which is partially overlapped with that underlying subconscious processing of threat-related signals. This also raises the possibility that the size overestimation and the pupil constrictions in response to the subconscious impending collision are two sides of the same coin, and their functional relationship merits further investigation.

In summary, our results demonstrate that when the observers could not discriminate any difference between the collision and the near-miss spheres, the sphere on the collision course looked larger and elicited greater pupil constrictions than that on the near-miss course. Moreover, the magnitude of size overestimation was comparable to that in the threat-perceived condition. Our findings highlight the functional dissociation between the pupillary response and the conscious perception of threat, and imply the existence of the subcortical threat-related visual pathway in the human brain.

## Author Contributions

LC and YJ conceived and designed the study. LC conducted the research and analyzed the data. LC wrote the manuscript, and XY, QX, YW, and YJ provided critical revisions.

## Conflict of Interest Statement

The authors declare that the research was conducted in the absence of any commercial or financial relationships that could be construed as a potential conflict of interest.
